# Ferroptosis in Podocytes: An Emerging Focus in Kidney Diseases

**DOI:** 10.3390/biology14121679

**Published:** 2025-11-26

**Authors:** Jun Feng, Yiqiong Ma, Chunyun Zhang

**Affiliations:** 1 Department of Geriatrics, Union Hospital, Tongji Medical College, Huazhong University of Science and Technology, Wuhan 430022, China; 2 Department of Nephrology, Shenzhen Hospital, Southern Medical University, Shenzhen 518101, China; yiqiongma@smu.edu.cn; 3 Department of Nephrology, Union Hospital, Tongji Medical College, Huazhong University of Science and Technology, Wuhan 430022, China

**Keywords:** ferroptosis, podocyte, kidney diseases, molecular mechanisms, treatment

## Abstract

Ferroptosis is a mechanism of programmed cell death that is characterized by iron-dependent lipid peroxidation, and it is closely associated with organismal metabolism. Modulating ferroptosis may offer therapeutic potential for various diseases. Podocytes are essential intrinsic cells in the renal corpuscle, and podocyte injury contributes to the progression of kidney diseases. A growing number of studies have confirmed that ferroptosis can occur in podocytes, and inhibiting podocyte ferroptosis may provide protective effects in multiple kidney diseases. However, the specific pathogenic molecular mechanisms and treatments for podocyte ferroptosis are still under investigation. In this review, we systematically review the core mechanisms of ferroptosis, including iron metabolism, lipid metabolism, and amino acid metabolism; summarize the molecular mechanisms of podocyte ferroptosis in various types of kidney disease; and discuss the current therapeutic strategies targeting podocyte ferroptosis. By synthesizing this information, we explore the potential of podocyte ferroptosis as a therapeutic target for kidney diseases, aiming to provide insights for both basic research and the clinical treatment of podocyte injury.

## Simple Summary

Ferroptosis is a type of programmed cell death that is characterized by iron-dependent lipid peroxidation and metabolic disorder. The roles of ferroptosis in the occurrence and progression of kidney diseases have attracted extensive attention, with recent studies also identifying an important role for podocyte ferroptosis. The abnormal metabolism of iron, lipids and amino acids is involved in the pathological process of ferroptosis, and the molecular mechanisms of podocyte ferroptosis are being investigated in various types of kidney disease. Current therapeutic strategies targeting podocyte ferroptosis provide protective effects in multiple kidney diseases, demonstrating that podocyte ferroptosis may serve as a key phenomenological event. Inhibiting podocyte ferroptosis may therefore provide a strategy for the treatment of kidney diseases.

## 1. Introduction

Ferroptosis, a distinct form of regulated cell death, was first described in 2012 [1]. The hallmark of ferroptosis is the accumulation of iron-dependent lipid hydroperoxides, which ultimately leads to cell death. Ferroptosis is characterized by mitochondrial atrophy, increased mitochondrial membrane density, loss of cristae structure, and rupture of the outer mitochondrial membrane without exhibiting the typical morphological or biochemical features of other forms of cell death, such as cell shrinkage, mitochondrial fragmentation, or nuclear condensation. Notably, there is no observable change in the cell membrane or chromatin morphology [2]. Ferroptosis has been associated with organ damage and degenerative pathological changes in various diseases, including diabetes, tumors, neurodegenerative disorders, cardiovascular and cerebrovascular diseases, ischemia–reperfusion injury and fibrosis [3,4,5,6,7,8]. Inhibiting ferroptosis has been shown to effectively slow disease progression. Currently, ferroptosis remains an active research area.

An increasing body of evidence indicates that ferroptosis plays a critical role in the progression of kidney diseases, encompassing acute kidney injury (AKI) from diverse etiologies, diabetic kidney disease (DKD), lupus nephritis (LN), renal tumors and renal fibrosis [9,10,11,12,13]. However, most studies have predominantly focused on ferroptosis in renal tubular cells, while overlooking the significance of ferroptosis in podocytes, an intrinsic cell in the renal corpuscle.

Podocytes, which is also called visceral epithelium of the renal corpuscle, are integral components of the glomerular filtration barrier that is essential for normal renal function. Podocyte damage and loss can result in proteinuria; thus, alleviating podocyte injury may reduce proteinuria development and exert a protective effect on renal function [14]. With the growing understanding of ferroptosis and increasing attention being paid to podocytes, the role of ferroptosis in podocyte injury is actively being investigated.

In this review, we comprehensively summarize the regulatory mechanisms underlying podocyte ferroptosis across different types of kidney disease and explore potential therapeutic strategies targeting podocyte ferroptosis with the aim of providing a theoretical foundation and guidance for the development of targeted therapies for podocytopathy.

## 2. Molecular Mechanisms of Ferroptosis

### 2.1. Iron Metabolism

**Figure 1:**
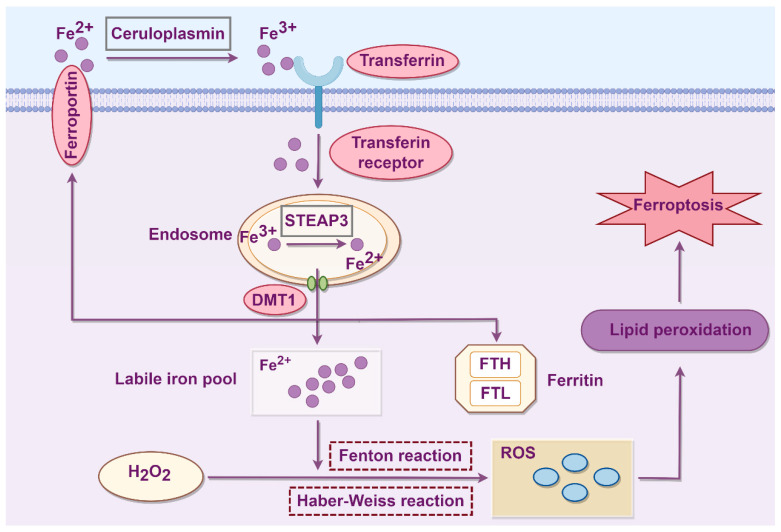
The process of abnormal iron metabolism leading to podocyte ferroptosis. STEAP3: Six-transmembrane epithelial antigen of prostate 3; DMT1: Divalent metal transporter 1; FTH: Ferritin heavy chain; FTL: Ferritin light chain; ROS: Reactive oxygen species (hydrogen peroxide, hydroxyl radicals, superoxide anions…).

Iron overload is a defining characteristic of ferroptosis, and iron accumulation induced by abnormal iron metabolism plays a pivotal role in the process of ferroptosis. The disruption of the balance between iron uptake, storage, utilization and excretion promotes lipid peroxidation via Fenton and Haber-Weiss reactions, thereby contributing to ferroptosis initiation [15]. The process of abnormal iron metabolism leading to ferroptosis is shown in Figure 1. Cellular iron homeostasis is maintained through an intricate iron transport system, where ceruloplasmin catalyzes the oxidation of Fe^2+^ derived from red blood cell degradation and intestinal absorption into Fe^3+^, which subsequently binds to transferrin (TF) on the cell membrane to form TF-Fe^3+^. TF-Fe^3+^ is internalized into endosome via the transferrin receptor (TFRC). Then, Fe^3+^ is reduced to Fe^2+^ by iron reductase and six-transmembrane epithelial antigen of prostate 3 (STEAP3). Fe^2+^ in the endosomal lumen is transported to the labile iron pool (LIP) and ferritin in the cytoplasm via divalent metal transporter 1 (DMT1). Ferroportin (FPN), an iron efflux membrane protein, mediates the export of excess Fe^2+^ out of cells.

The expression levels of genes and proteins involved in iron metabolism can modulate cellular sensitivity to ferroptosis. Ferritin, composed of a heavy chain (FTH) and light chain (FTL), primarily functions in iron storage and oxidation, with FTH playing a dominant role in these processes [16]. The inhibition of iron metabolism-related proteins such as TFRC, FTH and FTL can suppress ferroptosis [17]. Further investigation into the cellular and molecular mechanisms underlying iron metabolism may enhance our understanding of the fundamental principles governing ferroptosis.

### 2.2. Lipid Metabolism

**Figure 2:**
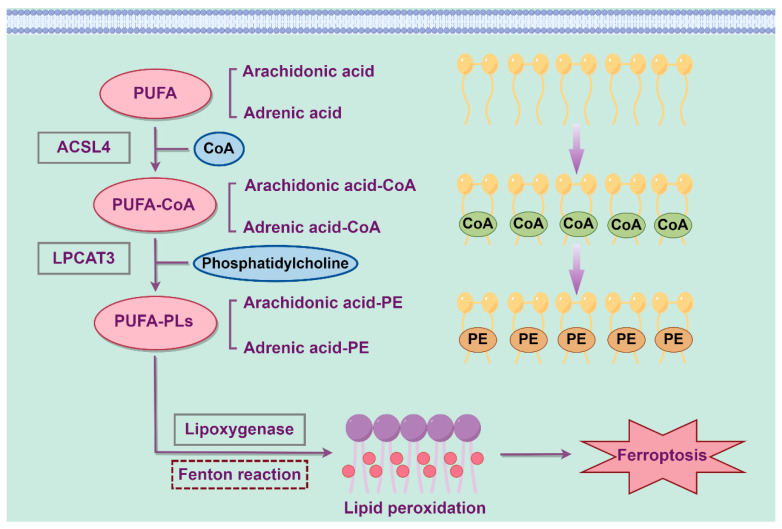
The process of abnormal lipid metabolism leading to ferroptosis. PUFA: Polyunsaturated fatty acid; ACSL4: Acyl-CoA synthetase long-chain family member 4; LPCAT3: Lysophosphatidylcholine acyltransferase 3; PL: Phospholipid; PE: Phosphatidylethanolamine.

Lipid molecules in cells serve multiple critical functions, including signal transduction, energy storage and as constituents of biological membranes. Polyunsaturated fatty acids (PUFAs), which are components of phospholipids prone to lipid peroxidation during ferroptosis, influence the extent of lipid peroxidation within cells and determine cellular sensitivity to ferroptosis [18]. Lipid peroxidation is closely associated with ferroptosis and can be initiated by the Fenton reaction and lipoxygenases. Genes involved in fatty acid metabolism, such as acyl-CoA synthetase long-chain family member 4 (ACSL4), and genes involved in lipid remodeling, such as lysophosphatidylcholine acyltransferase 3 (LPCAT3), have been identified as key biomarkers of ferroptosis. ACSL4 plays a pivotal role in activating long-chain unsaturated fatty acids, such as arachidonic acid (AA) and adrenic acid (AdA), for fatty acid oxidation and lipid biosynthesis [19]. Additionally, studies have demonstrated that ACSL4 mediates phospholipid metabolism by catalyzing the formation of PUFA-CoA and contributes to ferroptosis [20]. Research has shown that the knockout of ACSL4 inhibits ferroptosis, whereas the overexpression of ACSL4 modulates cellular lipid composition and enhances sensitivity to ferroptosis [21]. PUFA-CoA is then converted by lysophosphatidylcholine acyltransferase 3 (LPCAT3) into PUFA-containing phospholipids (PUFA-PLs), which is oxidized by lipoxygenase to confer lipid peroxidation [22]. Phosphatidylethanolamine (PE) containing AA or AdA serves as a critical phospholipid that induces ferroptosis in cells. Increasing the expression of ACSL4 and LPCAT3 or enhancing the activity of lipoxygenases results in the accumulation of lipid peroxides, ultimately leading to ferroptosis [23]. The process of abnormal lipid metabolism leading to ferroptosis is shown in Figure 2.

### 2.3. Amino Acid Metabolism

**Figure 3:**
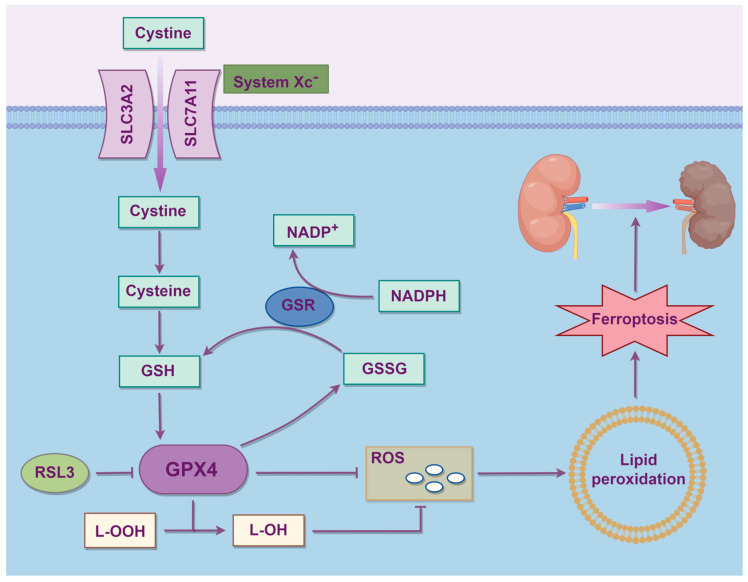
The process of aberrant amino acid metabolism leading to ferroptosis. SLC7A11: Solute carrier family 7 member 11; SLC3A2: Solute carrier family 3 member 2; System Xc^−^: Cystine-glutamate antiporter; GSH: Glutathione; GPX4: Glutathione peroxidase 4; GSSG: Glutathione disulfide; GSR: Glutathione reductase; L-OOH: lipid hydroperoxides; L-OH: lipid alcohols; RSL3: RAS-selective lethal 3; ROS: Reactive oxygen species.

The cystine-glutamate antiporter (system Xc^−^) is a heterodimeric amino acid transporter located on the cell membrane composed of solute carrier family 7 member 11 (SLC7A11) and solute carrier family 3 member 2 (SLC3A2), and it serves as a key hub in regulating ferroptosis. System Xc^−^ mainly maintains redox homeostasis by importing cystine, which is then converted into cysteine for the synthesis of the antioxidant glutathione (GSH). The conversion of cystine into cystine is the rate-limiting step in GSH synthesis. By regulating system Xc^−^, the levels of cysteine and GSH synthesis in cells can be controlled, further influencing ferroptosis. Some drugs, including the ferroptosis inducer Erastin, extracellular glutamate, sorafenib and sulfasalazine, can block the system Xc^−^ signaling pathway and trigger ferroptosis [24]. Glutathione peroxidase 4 (GPX4) mainly maintains cellular redox homeostasis by converting free hydrogen peroxide into water, reducing lipid hydroperoxides (L-OOH) into lipid alcohols (L-OH), with the concomitant oxidation of GSH into glutathione disulfide (GSSG). Both processes are crucial for maintaining cellular redox homeostasis and can block reactive oxygen species (ROS)-induced reactions, interrupting ferroptosis caused by harmful lipid peroxides. Inhibiting GPX4 leads to the accumulation of lipid peroxides, thereby triggering ferroptosis [25]. RAS-selective lethal 3 (RSL3) inhibits GPX4 and triggers ferroptosis, but not by blocking system Xc^−^ [26]. Regulating the system Xc^−^-GSH-GPX4 axis to promote or inhibit ferroptosis for disease treatment remains a current research hotspot. The process of aberrant amino acid metabolism leading to ferroptosis is shown in Figure 3.

## 3. Podocyte Ferroptosis in Kidney Diseases

### 3.1. Diabetic Kidney Disease

Diabetic kidney disease represents the most prevalent and severe microvascular complication of diabetes and is among the leading causes of end-stage renal disease [27]. Proteinuria, a DKD hallmark, serves as a critical indicator of DKD prognosis. Podocyte injury is closely associated with proteinuria development. Our prior research demonstrated that mitochondrial dysfunction in podocytes contributes to podocyte apoptosis and proteinuria formation in DKD, and enhancing mitochondrial recovery can effectively mitigate podocyte apoptosis and proteinuria [28].

**Figure 4:**
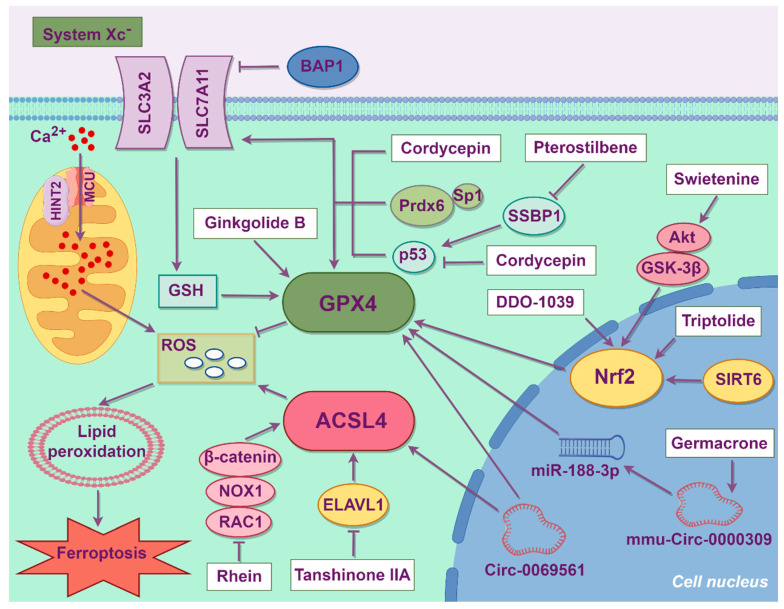
The molecular mechanisms of podocyte ferroptosis and action mechanisms of selected Chinese herbal medicine in DKD. ACSL4: Acyl-CoA synthetase long-chain family member 4; Akt: Protein kinase B; BAP1: Brca1-associated protein 1; ELAVL1: Embryonic lethal abnormal visual-like protein 1; GPX4: Glutathione peroxidase 4; GSH: Glutathione; GSK-3β: Glycogen synthase kinase 3β; HINT2: Histidine triad nucleotide-binding protein 2; MCU: Mitochondrial calcium uniporter; NOX1: NADPH oxidase 1; Nrf2: Nuclear factor erythroid 2-related factor 2; Prdx6: Peroxiredoxin 6; Rac1: Ras-related C3 botulinum toxin substrate 1; ROS: Reactive oxygen species; SIRT6: Sirtuin 6; SLC3A2: Solute carrier family 3 member 2; SLC7A11: Solute carrier family 7 member 11; Sp1: Specificity protein 1; SSBP1: Single-strand DNA-binding protein 1.

Current evidence suggests that renal hemodynamics, metabolic disturbances, inflammatory responses, aging processes, epigenetic regulation, epithelial–mesenchymal transition, and cell death pathways may contribute to podocyte damage [29]. With the intensifying focus on ferroptosis, recent studies have revealed that ferroptosis also plays a pivotal role in podocyte damage in DKD. The mechanisms of podocyte ferroptosis in DKD (Figure 4) remain under investigation.

Exposure to high glucose concentrations decreases cystine uptake, GSH content and SLC7A11 expression in podocytes, thereby promoting oxidative stress and ferroptosis activation. Overexpression of SLC7A11 can ameliorate cystine intake and alleviate oxidative stress. Mechanically, high glucose levels promote brca1-associated protein 1 (BAP1) expression, which is a tumor suppressor. BAP1 can target SLC7A11, reduce the H2Aub occupancy on the SLC7A11 promoter, inhibit the function of the amino acid transport complex system Xc^−^, and further lead to lipid peroxidation and ferroptosis in podocytes [30]. The recently discovered antioxidant enzyme peroxiredoxin 6 (Prdx6) was observed to downregulate ferroptosis in various cancer cell lines, such as H1299, A549 and 293FT cells [31]. Exposure of podocytes to high glucose concentrations reduces the expression of Prdx6 and specificity protein 1 (Sp1). Overexpression of Prdx6 upregulates SLC7A11 and GPX4 expression, inhibits the generation of ROS and malondialdehyde (MDA), a product of lipid peroxidation, and restores superoxide dismutase (SOD) activity and GSH content. Sp1 binding to its three binding motifs in the Prdx6 promoter activated Prdx6 gene expression, leading to increased Prdx6 enzyme activity, thereby reducing oxidative stress and ferroptosis in DKD podocytes [32].

In DKD patients, Circ-0069561, a novel diagnostic marker of DKD progression, is significantly upregulated in the renal glomeruli of DKD patients, and its level is positively correlated with the formation of massive proteinuria. Functional network analysis revealed the correlation between Circ-0069561 level and ferroptosis, while the downregulation of Circ-0069561 expression can alleviate high glucose-induced podocyte ferroptosis [33].

The mitochondria are the main source of ROS. Therefore, mitochondrial dysfunction is also associated with the induction of ferroptosis to some extent [34]. High-glucose stimulation leads to a decline in antioxidant capacity in podocytes, increasing ROS formation and lipid peroxidation. Sirtuin 6 (SIRT6) can reduce cellular oxidative stress, inflammatory responses and renal fibrosis, thereby maintaining cellular homeostasis and delaying the progression of kidney diseases [35,36]. SIRT6 protects podocytes from ferroptosis by enhancing mitochondrial membrane potential and preserving mitochondrial morphology by activating nuclear factor erythroid 2-related factor 2 (Nrf2)/GPX4 expression [37]. Histidine triad nucleotide-binding protein 2 (HINT2) modulates calcium dynamics by regulating the mitochondrial calcium uniporter (MCU) complex [38]. HINT2 binding to the MCU promotes ubiquitination and proteasomal degradation of the latter. Consequently, downregulation of HINT2 in DKD podocytes upregulates MCU, mitochondrial Ca^2+^ influx and ROS production, culminating in ferroptosis. HINT2 inhibition might therefore be a feasible strategy to alleviate podocyte damage and treat DKD [39].

The regulation of immune status in DKD kidneys is closely related to ferroptosis. Autophagy, apoptosis and complement activation may be involved in the ferroptosis process, and the disorder of the immune microenvironment further aggravates ferroptosis [40,41]. The upregulation of the ferroptosis marker CD44 and the downregulation of zinc finger protein 36 are associated with the increased polarization of renal M1 macrophages and the formation of inflammation in high glucose-treated podocytes [42].

### 3.2. Acute Kidney Injury

The pathophysiology of AKI involves multiple cellular mechanisms that can damage kidneys and disrupt the balance of fluids, electrolytes and waste products [43]. Podocytes are significantly affected during the onset of AKI, and podocyte injury is associated with AKI progression [44]. Reportedly, ferroptosis is a mechanism of podocyte injury in AKI.

RNA sequencing analysis indicated that significant changes occur in the oxidative stress and lipid metabolism signaling pathways related to ferroptosis in the kidneys of mice subjected to cisplatin-induced AKI. Podocyte ferroptosis activation is manifested as intracellular iron overload, increased production of MDA and ROS and upregulated expression of chemokine fractalkine (CX3CL1), an inflammatory chemokine. The inhibition of CX3CL1 can effectively alleviate podocyte ferroptosis and endoplasmic reticulum stress, as well as reducing renal inflammation and macrophage infiltration [45]. The serum level of cytokine-induced inhibitor 1 (CIAPIN1), a vital anti-apoptotic protein, is decreased in septic AKI patients. CIAPIN1 expression was also downregulated in podocytes cultured with lipopolysaccharide. CIAPIN1 overexpression increases phosphatidylinositol 3-kinase (PI3K)/protein kinase B (Akt) phosphorylation, which alleviates podocyte ferroptosis, while the PI3K/Akt pathway inhibitor LY294002 can reverse this protective effect. Therefore, CIAPIN1 inhibits ferroptosis of podocytes cultured with LPS through the PI3K/Akt signaling pathway [46].

### 3.3. Hepatitis B Virus-Associated Glomerulonephritis

Viral infection, a common cause of kidney disease, injures kidneys primarily through two processes: systemic inflammation and the direct infection of kidney cells [47]. To delineate the mechanism of virus-induced podocyte injury, RNA sequencing was performed in lentivirus-infected podocytes. That study demonstrated the activation of the ferroptosis related pathway and that the innate immune response results in the production of interferons and other cytokines which act on podocytes in an autocrine manner to induce ferroptosis [48].

Hepatitis B virus associated-glomerulonephritis (HBV-GN), which remains a huge healthcare burden worldwide, has been identified as the most common extrahepatic lesion caused by HBV infections [49]. Podocyte injury may be an important factor in the pathogenesis of HBV-GN [50]. The HBV X (HBx) protein encoded by HBV modulates multiple pathways, including HBV replication, transcription, the cell cycle and DNA repair [51]. HBx induces oxidative stress and lipid peroxidation, which are integral to the process of ferroptosis [52]. Podocytes transfected with HBx experience ferroptosis, which is manifested by the downregulation of GPX4 and SLC7A11, upregulation of ACSL4, and increased intracellular iron, MDA and ROS levels [53].

### 3.4. Lupus Nephritis

Lupus nephritis is a common and severe complication of systemic lupus erythematosus (SLE). The pathogenesis of LN is complex, involving the initiation of disease by immune complexes, the activation of immune responses within the kidney and the reaction of renal parenchymal cells to this damage [54]. Podocyte injury is a common feature of kidney autoimmune diseases, including LN, contributing to kidney dysfunction [55]. Ovarian tumor domain-containing ubiquitin aldehyde binding protein 1 (OTUB1), a deubiquitinating enzyme, has emerged as a potential therapeutic target due to its role in cellular protection and the regulation of ferroptosis, which is linked to LN. Decreased OTUB1 expression in the podocytes of LN patients is corelated with the severity of disease. A deficiency of OTUB1 mediates the downregulation of SLC7A11 expression, thereby promoting ferroptosis [56].

### 3.5. Focal Segmental Glomerulosclerosis

Focal segmental glomerulosclerosis (FSGS) is a common pathological lesion in primary glomerular diseases, and podocytes are the main target cells [57]. GPX4 expression is reduced in the podocytes of FSGS patients and Adriamycin (ADR)-challenged mice, implicating podocyte ferroptosis in FSGS. The inhibition of ferroptosis with Ferrostatin-1 ameliorates the GPX4 expression suppression, podocyte injury, proteinuria, glomerulosclerosis and tubulointerstitial fibrosis in ADR-induced nephropathy [58]. Thus, inhibiting ferroptosis may represent a potential therapy for FSGS.

### 3.6. Fabry Disease

Fabry disease (FD) results from pathogenic galactosidase A (GLA) variants, leading to a deficiency in lysosomal α-galactosidase A (α-Gal A) and accumulation of the sphingolipid globotriaosylceramide (Gb3). Podocyte injury is an early hallmark of Fabry nephropathy, where Gb3 accumulation contributes to increased extracellular matrix synthesis and fibrosis [59]. However, the mechanism underlying Gb3-induced cell dysfunction remains largely unknown. Podocytes from induced pluripotent stem cells (iPSCs) generated from patients with FD display reduced α-Gal A activity and Gb3 accumulation. Proteomic profiling has revealed that the ferroptosis-associated protein arachidonate 15-lipoxygenase is the most upregulated protein in FD podocytes while ferroptosis is the most enriched pathway, demonstrating that podocyte ferroptosis plays a key role in the pathological mechanism of FD [60].

### 3.7. Cystinosis

Cystinosis, a rare and incurable lysosomal storage disease, is caused by mutation of the CTNS gene encoding the cystine transporter cystinosin [61]. Cystinosis patients may display podocyte loss in the early stages, followed by proteinuria and further FSGS lesions. In cystinosis, podocytes exhibit mitochondrial dysfunction characterized by mitochondrial fragmentation, an impaired tricarboxylic acid cycle, metabolic abnormalities, and increased mitochondrial ROS production and lipid peroxidation, ultimately leading to podocyte ferroptosis and detachment [62].

## 4. Treatment Strategies of Ferroptosis

**Figure 5:**
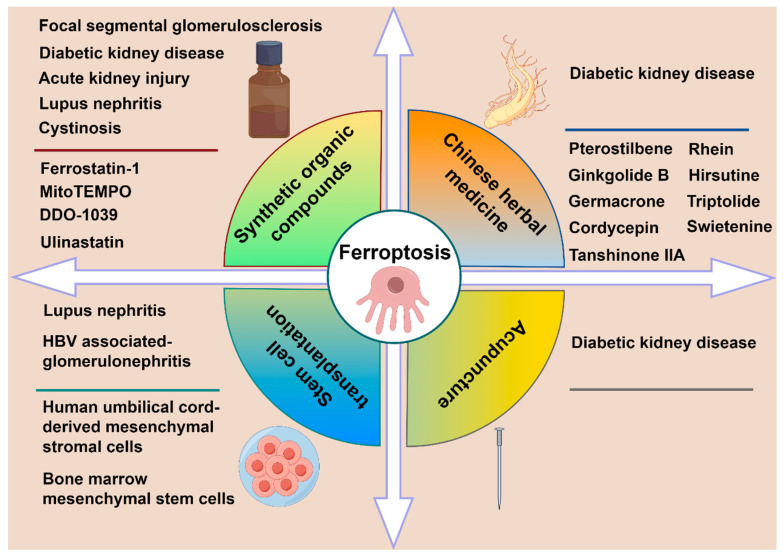
The treatment approaches for podocyte ferroptosis in different kinds of kidney diseases.

Based on current therapeutic advances in podocyte ferroptosis, we categorize the treatment approaches into four main types, namely synthetic organic compounds, stem cell transplantation, Chinese herbal medicine and acupuncture therapy. Different treatment regimens are applied to distinct kidney diseases, as illustrated in Figure 5. Table 1 lists the specific therapeutic mechanisms and effects.

### 4.1. Synthetic Organic Compounds

Synthetic organic compounds remain the most longstanding and convenient treatment option in current clinical therapy. Numerous synthetic organic compounds exert anti-ferroptosis effects through multiple signaling pathways, thereby producing therapeutic functions in different kinds of disease, including podocyte ferroptosis.

As Nrf2 is a key transcription factor regulating cellular response to oxidative stress, Nrf2-activated gene expression is an attractive therapeutic target for DKD and is also critically involved in ferroptosis regulation [63]. DDO-1039, a novel small-molecule Nrf2 activator, significantly increases Nrf2 expression and Nrf2 nuclear translocation, which upregulates GPX4 expression to inhibit ferroptosis in podocytes of diabetic mice [64].

Ulinastatin, known as an antioxidant and anti-inflammatory, upregulates SLC7A11 expression by reducing miR-144-3p in lipopolysaccharide (LPS)-induced podocytes, reducing the accumulation of Fe^2+^ and lipid ROS and maintaining the cytoskeleton [65]. Cytochrome P450 substrate drugs, including rifampicin, promethazine, omeprazole, indole-3-carbinol, carvedilol, propranolol, estradiol, and thyroid hormones, scavenge lipid peroxidation radicals and mitigate lipid peroxidation in podocytes, tubular cells and renal fibroblasts, thereby exerting antiferroptotic properties in AKI [66]. Ferrostatin-1 is an established inhibitor of ferroptosis [67]. In OTUB1 knockout podocytes, Ferrostatin-1 treatment restores SLC7A11 expression, reduces MDA levels and increases cysteine and glutathione levels, suggesting that ferroptosis inhibition could be a therapeutic strategy for LN [56].

MitoTEMPO is a mitochondria-targeted antioxidant that scavenges mitochondrial ROS (mtROS) and blocks ROS-induced lipid peroxidation. MitoTEMPO reduces mtROS generation, thereby alleviating lipid peroxidation and suppressing podocyte ferroptosis, thus attenuating proteinuria formation in cystinosis [62].

### 4.2. Stem Cell Transplantation

Stem cell transplantation has demonstrated significant therapeutic potential in the field of regenerative medicine and has garnered increasing attention, offering novel and effective treatment approaches for various inflammatory diseases and degenerative disorders, including kidney diseases [68]. Mesenchymal stem cells (MSCs) have become the most commonly used cell type in cell therapy as they are found in various tissues such as bone marrow, umbilical cord and adipose tissue. The paracrine action of MSCs serves as their primary therapeutic mechanism; they exert healing effects through the secretion of many bioactive cytokines including anti-inflammatory factors, chemokines, growth factors and extracellular vesicles at injury sites [69].

Mesangial stem cell (MSC) transplantation has emerged as an alternative therapeutic strategy for ferroptosis in podocytes [70]. Bone marrow-derived MSCs carrying miR-223-3p inhibit histone deacetylase 2 expression can reduce signal transducer and activator of transcription 3 phosphorylation, maintain normal iron metabolism and effectively suppress lipid peroxidation in podocytes, thereby attenuating HBx-mediated ferroptosis [71]. MSC therapy has emerged as a viable therapeutic option for podocyte injury in LN [72]. Following MSC intervention, puromycin aminonucleoside-treated podocytes exhibit enhanced cytoskeletal stability and improved cellular viability. Mechanistically, MSCs attenuate podocyte ferroptosis by facilitating Nrf2 nuclear translocation and upregulating HO-1 and GPX4 expression [73].

### 4.3. Chinese Herbal Medicine

Traditional Chinese medicine (TCM) boasts a long history and has played a pivotal role in advancing global healthcare. Chinese herbal medicine formulas are chiefly composed of two or more medicinal compounds targeting relatively defined diseases, serving as primary therapeutic interventions in Chinese medical treatment [74]. In recent years, a growing number of researchers have focused on the therapeutic value of Chinese herbal medicines in the field of kidney diseases, particularly in DKD [75]. Figure 4 summarizes the mechanisms of different Chinese herbal medicines in the treatment of podocyte ferroptosis in DKD. Chinese herbal medicine treatments exert renal protective effects through multiple signaling pathways, including the Nrf2, AMP-activated protein kinase (AMPK) and mammalian target of rapamycin (mTOR) pathways [76,77,78]. Recent research has revealed the ferroptosis-suppressing capabilities of Chinese herbal medicines in podocytes.

Triptolide ameliorates proteinuria in diabetic mice by suppressing podocyte ferroptosis through the Nrf2 pathway. In podocytes, triptolide upregulates GPX4, FTH1, SLC7A11 and Nrf2 signaling while downregulating TFRC, thereby inhibiting the pathogenic transition of slit diaphragms to tight junctions, a hallmark of glomerular filtration barrier injury [79]. Hirsutine, a monomeric alkaloid extracted from the herb Uncaria, has a wide range of biological effects, including antioxidant, anti-proliferative and anti-apoptotic properties [80]. Hirsutine reduces hyperglycemia and insulin resistance in diabetic mice [81]. Hirsutine reduces the renal iron content and ROS and MDA levels while upregulating GPX4 and downregulating p53 expression in the podocytes of diabetic mice, thereby alleviating podocyte loss. Administration of the p53 agonist Nutlin-3 reverses the renoprotective effects of Hirsutine, demonstrating that Hirsutine mitigates DKD podocyte ferroptosis via the p53/GPX4 signaling pathway [82]. Ginkgolide B alleviates podocyte ferroptosis and oxidative stress, thereby exerting therapeutic effects against DKD. It achieves this by inhibiting GPX4 ubiquitination, which upregulates the expression of GPX4 and FTH1 while simultaneously suppressing TFRC expression, intracellular iron content and ROS formation [83]. Germacrone alleviates podocyte apoptosis in DKD, thereby exerting a protective effect on the kidneys. Germacrone suppresses the expression of ferroptosis-related proteins, mitigates mitochondrial damage and ROS production, and inhibits ferroptosis in DKD podocytes via the mmu_circRNA_0000309/miR-188-3p/GPX4 signaling pathway [84].

Proteomic analysis revealed that mitochondrial single-strand DNA-binding protein 1 (SSBP1) expression is significantly upregulated in the glomeruli of high fructose-fed rats, where it participates in promoting podocyte ferroptosis. Mechanistically, SSBP1 can bind to DNA-dependent protein kinase (DNA-PK) and p53. By activating DNA-PK, SSBP1 promotes p53 phosphorylation and its nuclear accumulation, which further suppresses SLC7A11 expression and promotes ferroptosis. Pterostilbene effectively alleviates high fructose-induced podocyte ferroptosis by downregulating SSBP1 and inhibiting the DNA-PK/p53 pathway [85]. Cordycepin suppresses HG-induced podocyte ferroptosis and ameliorates renal inflammation in diabetic mice by activating the SLC7A11/GPX4 pathway [86].

In DKD podocytes, embryonic lethal abnormal visual-like protein 1 (ELAVL1), an RNA-binding post-transcriptional regulator, stabilizes ACSL4 mRNA to upregulate its expression and promote ferroptosis. Tanshinone IIA is one of the main components of the root of the red-rooted Salvia miltiorrhiza Bunge and inhibits ferroptosis through the ELAVL1/ACSL4 pathway [87].

In DKD, Rhein reduces the accumulation of ROS, MDA and Fe^2+^, and mitigates podocyte ferroptosis by suppressing the Ras-related C3 botulinum toxin substrate 1 (Rac1)/NADPH oxidase 1 (NOX1)/β-catenin axis [88]. Swietenine alleviates oxidative stress and ferroptosis in high glucose-treated podocytes. Predictive network pharmacology suggests that Swietenine exerts protective effects against DKD by targeting the PI3K-Akt signaling pathway. Molecular docking analysis further indicated a potential interaction between Swietenine and Akt, confirming that Swietenine inhibits high glucose-induced podocyte ferroptosis by activating the Akt/glycogen synthase kinase 3β (GSK-3β)/Nrf2 signaling pathway [89].

### 4.4. Acupuncture

Acupuncture, a cornerstone of TCM, demonstrates therapeutic potential for enhancing kidney function, reducing proteinuria, regulating hypertension and treating various renal conditions [90]. In diabetic rat models, the application of acupuncture therapy enhances antioxidant capacity, maintains iron homeostasis and inhibits epithelial–mesenchymal transition and ferroptosis in podocytes. Collectively, these effects improve podocyte structure, restore filtration function, reduce proteinuria, and prevent DKD progression [91].

**Table 1:** The specific molecular mechanisms and effects of different treatments in podocyte ferroptosis.

Treatment	Medicine/Cells	Disease	Model	Target	Signaling Pathway	Effects	Reference
Synthetic organic compounds	DDO-1039	DKD	Mice	Nrf2	Keap1/Nrf2	Oxidative stress ↓ GPX4 ↑	[64]
	Cytochrome P450 substrate drugs	AKI	Mice	-	-	Lipid peroxyl radicals ↓	[66]
	Ulinastatin	AKI	Mice/cell line	MiR-144-3p	MiR-144-3p/SLC7A11	Fe^2+^, ROS ↓	[65]
	Ferrostatin-1	LN	Mice	OTUB1	OTUB1/ SLC7A11	Fe^2+^, ROS, MDA ↓ SLC7A11/GSH ↑	[56]
	Ferrostatin-1	FSGS	Mice	GPX4	GPX4	GSH ↑	[58]
	MitoTEMPO	Cystinosis	Zebrafish	-	-	Mitochondrial function ↑ lipid peroxidation ↓	[62]
Stem cell transplantation	Bone marrow mesenchymal stem cells	HBV-GN	Human cell line	MiR-223-3p	HDAC2/STAT3	Fe^2+^, ROS, MDA ↓ GPX4/SLC7A11/ACSL4 ↑	[71]
	Human umbilical cord-derived mesenchymal stromal cells	LN	Mice	Nrf2	Nrf2/HO-1/GPX4	ROS, MDA ↓ SOD, GSH ↑	[73]
Chinese herbal medicine	Hirsutine	DKD	Mice	GPX4	p53/GPX4	Fe^2+^, ROS, MDA ↓ Mitochondrial morphology	[82]
	Triptolide	DKD	Mice	Nrf2	Nrf2	GPX4/FTH1/SLC7A11 ↑, mitochondrial function ↑, TFRC ↓, oxidative stress ↓	[79]
	Tanshinone IIA	DKD	Mouse cell line	ELAVL1	ELAVL1/ACSL4	Fe^2+^, ROS, MDA ↓ GSH ↑	[87]
	Rhein	DKD	Mice	Rac1	Rac1/NOX1/β-catenin	Fe^2+^, ROS, MDA ↓	[88]
	Pterostilbene	DKD	Rats	SSBP1	DNA-PK/p53	SLC7A11 ↑	[85]
	Germacrone	DKD	Mice	mmu_circRNA_0000309	miR-188-3p/GPX4	GPX4, mitochondrial function ↑	[84]
	Cordycepin	DKD	Mouse cell line	SLC7A11/GPX4	-	Fe^2+^, ROS, MDA ↓ GSH ↑	[86]
	Ginkgolide B	DKD	Mouse cell line	GPX4	GPX4	Fe^2+^, ROS, TFRC ↓ GPX4/FTH1 ↑	[83]
	Swietenine	DKD	Rats	Akt	Akt/GSK-3β/Nrf2	Fe^2+^, MDA, PTGS2 ↓ Oxidative stress ↓ GPX4/SLC7A11 ↑	[89]
Acupuncture		DKD	Rats	GPX4 and System Xc^−^	-	Oxidative stress ↓, iron homeostasis	[91]

ACSL4: Acyl-CoA synthetase long-chain family member 4; AKI: Acute kidney injury; Akt: Protein kinase B; DKD: Diabetic kidney disease; ELAVL1: Embryonic lethal abnormal visual-like protein 1; FSGS: Focal segmental glomerulosclerosis; FTH1: Ferritin heavy chain 1; GPX4: Glutathione peroxidase 4; GSH: Glutathione; GSK-3β: Glycogen synthase kinase 3β; HBV-GN: Hepatitis B virus-associated glomerulonephritis; HDAC2: Histone deacetylase 2; Keap1: Kelch-like ECH-associated protein 1; LN: Lupus nephritis; MDA: Malondialdehyde; NOX1: NADPH oxidase 1; Nrf2: Nuclear factor erythroid 2-related factor 2; OTUB1: Ovarian tumor domain-containing ubiquitin aldehyde binding protein 1; PTGS2: Prostaglandin-endoperoxide synthase 2; Rac1: Ras-related C3 botulinum toxin substrate 1; ROS: Reactive oxygen species; SLC7A11: Solute carrier family 7 member 11; SSBP1: Single-strand DNA-binding protein 1; STAT3: Signal transducer and activator of transcription 3; TFRC: Transferrin receptor. (↑, enhancement effect; ↓, attenuation effect).

## 5. Conclusions 

Studies investigating podocyte ferroptosis-induced kidney injury and its therapeutic strategies have proliferated rapidly over the past three years. Podocyte ferroptosis serves as a central pathological link connecting metabolic dysregulation, oxidative stress and kidney damage [92]. In this review, we consolidated the three major metabolic pathways implicated in ferroptosis, namely iron metabolism, lipid metabolism and amino acid metabolism. Podocyte ferroptosis has been observed in various kidney diseases, including DKD, HBV-GN, LN and AKI. The distinct regulatory mechanisms involved in podocyte ferroptosis across different types of kidney diseases within their specific pathological contexts remain to be fully explored. Current research has confirmed that the signaling pathways and key molecular targets related to podocyte ferroptosis primarily involve GPX4, system Xc^−^, ACSL4 and the Nrf2 signaling pathway [73,86]. Therapeutic strategies utilizing synthetic organic compounds, stem cell transplantation and TCM can exert anti-ferroptotic effects on podocytes in multiple kinds of kidney disease. Intervention strategies targeting GPX4, SLC7A11 and ACSL4 have also demonstrated preclinical efficacy.

## 6. Future Perspectives

Podocytes are essential structural elements of the glomerular filtration barrier but their unique architectures and terminally differentiated character impose significant challenges and limitations on ferroptosis research. This field consequently exhibits marked limitations and deficiencies. To overcome the aforementioned challenges and advance the development of podocyte-targeted ferroptosis therapeutics, it is imperative to integrate cutting-edge multidisciplinary technologies.

First, the limitations in dynamic monitoring techniques should be addressed. There is a critical lack of imaging technologies capable of specifically detecting key ferroptotic events, such as ROS bursts and lipid peroxide accumulation, within podocytes under in vivo conditions. Current methodologies, including chemical probes and biochemical assays, are inadequate for capturing the spatiotemporal dynamics of the ferroptosis process. Integrated multi-omics analysis, involving genomics, transcriptomics, proteomics, lipidomics and metabolomics, could be used to construct a predictive model and molecular subtyping system based on the key molecular features of ferroptosis [93]. Further exploring other indicators reflecting ferroptosis and combining artificial intelligence technology to construct dynamic analysis system represents a potential solution.

Second, current ferroptosis inhibitors universally lack podocyte-targeting specificity. Systemic administration not only risks reducing locally effective drug concentrations but also non-specifically interfering with the normal functions of other physiologically lipid peroxidation-dependent and ferroptosis-susceptible cells, leading to off-target effects and potential adverse consequences. There are some podocyte-specific proteins, which enables the development of targeted antibody or nucleotide-based therapeutics containing small fragments of DNA/RNA. Developing an intelligent drug delivery system based on engineered exosomes, nanocarriers or ligand-drug conjugates to achieve the efficient and specific delivery of ferroptosis-regulating drugs could help to maximize therapeutic efficacy while minimizing systemic side effects [94].

Third, disease heterogeneity is another limiting factor. In podocyte injury induced by different etiologies, the triggering mechanisms of ferroptosis, key regulatory molecules and downstream effector pathways may differ. This heterogeneity in disease background necessitates that researchers delve into the characteristics of ferroptosis within specific pathological contexts and develop personalized or disease subtype-specific intervention strategies. Humanized kidney organoid models can be used to simulate the occurrence of podocyte ferroptosis and its interactions with adjacent cells under various pathological stimuli in a highly simulated microenvironment.

Moreover, translational hurdles represent a major obstacle. Although ferroptosis inhibitors have demonstrated renoprotective potential in preclinical models, there is still no direct strategy for protecting against ferroptosis in current clinical practice. In future research, focus can be placed on developing synthetical ferroptosis inhibitors and extracts from Chinese herbal medicines, and applying them to clinical treatment through step-by-step clinical trials. Although their long-term safety, tolerability and efficacy in patients with kidney diseases remain to be fully evaluated, it still might be a feasible approach. Critical issues such as their pharmacokinetic properties, potential chronic toxicity and drug–drug interactions with other nephropathy treatments urgently require clarification. Strengthening translational medicine research to systematically evaluate the long-term benefits versus risks of podocyte ferroptosis-targeted therapies in clinical cohorts can promote the translation of this promising therapeutic strategy from basic research to clinical application.

Through continued in-depth research in these areas, targeted inhibition of podocyte ferroptosis may emerge as one of the key therapeutic approaches for kidney diseases in the future.

## Data Availability

No data was used for the research described in the article.

## Abbreviations

ACSL4	Acyl-CoA synthetase long-chain family member 4
AKI	Acute kidney injury
Akt	Protein kinase B
BAP1	Brca1-associated protein 1
DKD	Diabetic kidney disease
ELAVL1	Embryonic lethal abnormal visual-like protein 1
FSGS	Focal segmental glomerulosclerosis
FTH1	Ferritin heavy chain 1
GPX4	Glutathione peroxidase 4
GSH	Glutathione
GSK-3β	Glycogen synthase kinase 3β
HBV-GN	Hepatitis B virus-associated glomerulonephritis
HDAC2	Histone deacetylase 2
HINT2	Histidine triad nucleotide-binding protein 2
Keap1	Kelch-like ECH-associated protein 1
LN	Lupus nephritis
MCU	Mitochondrial calcium uniporter
MDA	Malondialdehyde
NOX1	NADPH oxidase 1
Nrf2	Nuclear factor erythroid 2-related factor 2
OTUB1	Ovarian tumor domain-containing ubiquitin aldehyde binding protein 1
Prdx6	Peroxiredoxin 6
PTGS2	Prostaglandin-endoperoxide synthase 2
Rac1	Ras-related C3 botulinum toxin substrate 1
ROS	Reactive oxygen species
SIRT6	Sirtuin 6
SLC3A2	Solute carrier family 3 member 2
SLC7A11	Solute carrier family 7 member 11
Sp1	Specificity protein 1
SSBP1	Single-strand DNA-binding protein 1
STAT3	Signal transducer and activator of transcription 3
TFRC	Transferrin receptor

## References

[1] Dixon S.J., Lemberg K.M., Lamprecht M.R., Skouta R., Zaitsev E.M., Gleason C.E., Patel D.N., Bauer A.J., Cantley A.M., Yang W.S. (2012). Ferroptosis: An iron-dependent form of nonapoptotic cell death. Cell.

[2] Zhan P., Lu X., Li Z., Wang W.J., Peng K., Liang N.N., Wang Y., Li J., Fu L., Zhao H. (2022). Mitoquinone alleviates bleomycin-induced acute lung injury via inhibiting mitochondrial ros-dependent pulmonary epithelial ferroptosis. Int. Immunopharmacol..

[3] Wei F., Ruan B., Dong J., Yang B., Zhang G., Yeung W.K.K., Wang H., Cao W., Wang Y. (2024). Asperosaponin vi inhibition of dnmt alleviates gpx4 suppression-mediated osteoblast ferroptosis and diabetic osteoporosis. J. Adv. Res..

[4] Nan P., Wang X., Li A., Ge Y., Gu Z., Wang Y., Tao R. (2025). Tspan15 sustains itgb1 stability to block gemcitabine-induced ferroptosis in pancreatic ductal adenocarcinoma through the fak/akt/mtor-gpx4 cascade. Redox Biol..

[5] Zha X., Liu X., Wei M., Huang H., Cao J., Liu S., Bian X., Zhang Y., Xiao F., Xie Y. (2025). Microbiota-derived lysophosphatidylcholine alleviates alzheimer’s disease pathology via suppressing ferroptosis. Cell Metab..

[6] Cao Y., Huang M.Y., Mao C.H., Wang X., Xu Y.Y., Qian X.J., Ma C., Qiu W.Y., Zhu Y.C. (2025). Structural changes in cerebral microvasculature induced by ferroptosis contribute to blood-brain barrier disruption in alzheimer’s disease: An autopsy study. Alzheimers Dement..

[7] Wang X., Chen T., Chen S., Zhang J., Cai L., Liu C., Zhang Y., Wu X., Li N., Ma Z. (2025). Sting aggravates ferroptosis-dependent myocardial ischemia-reperfusion injury by targeting gpx4 for autophagic degradation. Signal Transduct. Target. Ther..

[8] Wang J., Song Y., Tan X., Wang T., Shi Y., Xu X., Du J., Yu Z., Song B. (2025). Targeting pim1 by bruceine d attenuates skin fibrosis via myofibroblast ferroptosis. Redox Biol..

[9] Wei W., Yang L., Wang B., Tang L., Li J., Liu C., Huang Y., Zhang Z., Zhang D., Zhang L. (2025). Remote ischemic preconditioning attenuates mitochondrial dysfunction and ferroptosis of tubular epithelial cells by inhibiting nox4-ros signaling in acute kidney injury. Int. J. Biol. Sci..

[10] Tian S., Zhou S., Wu W., Lin Y., Wang T., Sun H., A-Ni-Wan A.S., Li Y., Wang C., Li X. (2025). Glp-1 receptor agonists alleviate diabetic kidney injury via beta-klotho-mediated ferroptosis inhibition. Adv. Sci..

[11] Cheng Q., Mou L., Su W., Chen X., Zhang T., Xie Y., Xue J., Lee P.Y., Wu H., Du Y. (2023). Ferroptosis of cd163^+^ tissue-infiltrating macrophages and cd10^+^ pc^+^ epithelial cells in lupus nephritis. Front. Immunol..

[12] Helleux A., Davidson G., Lallement A., Hourani F.A., Haller A., Michel I., Fadloun A., Thibault-Carpentier C., Su X., Lindner V. (2025). Tfe3 fusions drive oxidative metabolism and ferroptosis resistance in translocation renal cell carcinoma. EMBO Mol. Med..

[13] Guo Q., Chen J., Wu J., Mo Z., Ye L., Zhong W., Zhang Y., Lai H., Zhang Y., Qiu J. (2024). Bioprinted mesenchymal stem cell microfiber-derived extracellular vesicles alleviate unilateral renal ischemia-reperfusion injury and fibrosis by inhibiting tubular epithelial cells ferroptosis. Bioact. Mater..

[14] Li W., Zhang Z., Peng Z., Hu H., Cui X., Zhu Z., Qi Y., Chen W., Liu H., Liang W. (2025). Piezo1-mediated calcium signaling and podocyte injury in diabetic kidney disease. J. Am. Soc. Nephrol..

[15] Su S., Wang Y., Xiang Y., Mao C., Wang Y., Huang J., Liu X., Wang C., Liu H., Li Z. (2025). A smart hydrogel dressing balances reactive oxygen species levels for effective treatment of bacteria-infected atopic dermatitis. Adv. Healthc. Mater..

[16] Chen Y., Zhang J., Tian Y., Xu X., Wang B., Huang Z., Lou S., Kang J., Zhang N., Weng J. (2024). Iron accumulation in ovarian microenvironment damages the local redox balance and oocyte quality in aging mice. Redox Biol..

[17] Zhong P., Li L., Feng X., Teng C., Cai W., Zheng W., Wei J., Li X., He Y., Chen B. (2024). Neuronal ferroptosis and ferroptosis-mediated endoplasmic reticulum stress: Implications in cognitive dysfunction induced by chronic intermittent hypoxia in mice. Int. Immunopharmacol..

[18] Wang Y., Hu M., Cao J., Wang F., Han J.R., Wu T.W., Li L., Yu J., Fan Y., Xie G. (2025). Acsl4 and polyunsaturated lipids support metastatic extravasation and colonization. Cell.

[19] Samovich S.N., Mikulska-Ruminska K., Dar H.H., Tyurina Y.Y., Tyurin V.A., Souryavong A.B., Kapralov A.A., Amoscato A.A., Beharier O., Karumanchi S.A. (2024). Strikingly high activity of 15-lipoxygenase towards di-polyunsaturated arachidonoyl/adrenoyl-phosphatidylethanolamines generates peroxidation signals of ferroptotic cell death. Angew. Chem. Int. Ed. Engl..

[20] Doll S., Proneth B., Tyurina Y.Y., Panzilius E., Kobayashi S., Ingold I., Irmler M., Beckers J., Aichler M., Walch A. (2017). Acsl4 dictates ferroptosis sensitivity by shaping cellular lipid composition. Nat. Chem. Biol..

[21] Huang Q., Ru Y., Luo Y., Luo X., Liu D., Ma Y., Zhou X., Linghu M., Xu W., Gao F. (2024). Identification of a targeted acsl4 inhibitor to treat ferroptosis-related diseases. Sci. Adv..

[22] Shahtout J.L., Eshima H., Ferrara P.J., Maschek J.A., Cox J.E., Drummond M.J., Funai K. (2024). Inhibition of the skeletal muscle lands cycle ameliorates weakness induced by physical inactivity. J. Cachexia Sarcopenia Muscle.

[23] Cui J., Wang Y., Tian X., Miao Y., Ma L., Zhang C., Xu X., Wang J., Fang W., Zhang X. (2023). Lpcat3 is transcriptionally regulated by yap/zeb/ep300 and collaborates with acsl4 and yap to determine ferroptosis sensitivity. Antioxid. Redox Signal..

[24] Du Y., Guo Z. (2022). Recent progress in ferroptosis: Inducers and inhibitors. Cell Death Discov..

[25] Ye Z., Cheng M., Lian W., Leng Y., Qin X., Wang Y., Zhou P., Liu X., Peng T., Wang R. (2025). Gpx4 deficiency-induced ferroptosis drives endometrial epithelial fibrosis in polycystic ovary syndrome. Redox Biol..

[26] Zhang F., Xiao Y., Huang Z., Wang Y., Wan W., Zou H., Wang B., Qiu X., Yang X. (2024). Upregulation of gpx4 drives ferroptosis resistance in scleroderma skin fibroblasts. Free. Radic. Biol. Med..

[27] Petrica L. (2024). Special issue ijms-molecular mechanisms of diabetic kidney disease. Int. J. Mol. Sci..

[28] Feng J., Chen Z., Ma Y., Yang X., Zhu Z., Zhang Z., Hu J., Liang W., Ding G. (2022). Akap1 contributes to impaired mtdna replication and mitochondrial dysfunction in podocytes of diabetic kidney disease. Int. J. Biol. Sci..

[29] Defronzo R.A., Reeves W.B., Awad A.S. (2021). Pathophysiology of diabetic kidney disease: Impact of sglt2 inhibitors. Nat. Rev. Nephrol..

[30] Peiyao R., Xueli M., Wenbo S., Danna Z., Jianguang G., Juan J., Qiang H. (2025). High glucose induces podocyte ferroptosis through bap1/slc7a11 pathway. Heliyon.

[31] Lu B., Chen X.B., Hong Y.C., Zhu H., He Q.J., Yang B., Ying M.D., Cao J. (2019). Identification of prdx6 as a regulator of ferroptosis. Acta Pharmacol. Sin..

[32] Zhang Q., Hu Y., Hu J.E., Ding Y., Shen Y., Xu H., Chen H., Wu N. (2021). Sp1-mediated upregulation of prdx6 expression prevents podocyte injury in diabetic nephropathy via mitigation of oxidative stress and ferroptosis. Life Sci..

[33] Chen C., Liu X., Zhu S., Wang Y., Ma Y., Hu Z., Wu Y., Jiang L. (2025). Circ-0069561 as a novel diagnostic biomarker for progression of diabetic kidney disease. Ren. Fail..

[34] Yang J., Lu X., Hao J.L., Li L., Ruan Y.T., An X.N., Huang Q.L., Dong X.M., Gao P. (2025). Vstm2l protects prostate cancer cells against ferroptosis via inhibiting vdac1 oligomerization and maintaining mitochondria homeostasis. Nat. Commun..

[35] Yang X., Feng J., Liang W., Zhu Z., Chen Z., Hu J., Yang D., Ding G. (2021). Roles of sirt6 in kidney disease: A novel therapeutic target. Cell. Mol. Life Sci..

[36] Hao Y., Hu J., Zhang Z., Guan Q., Wang J., Tao Y., Cheng J., Fan Y. (2025). Sirt6 deficiency exacerbates angiotensin ii-induced lipid nephrotoxicity by affecting pld6-derived cardiolipin metabolism in podocytes. Cell. Signal..

[37] Du L., Guo C., Zeng S., Yu K., Liu M., Li Y. (2024). Sirt6 overexpression relieves ferroptosis and delays the progression of diabetic nephropathy via nrf2/gpx4 pathway. Ren. Fail..

[38] Li S., Chen J., Liu M., Chen Y., Wu Y., Li Q., Ma T., Gao J., Xia Y., Fan M. (2021). Protective effect of hint2 on mitochondrial function via repressing mcu complex activation attenuates cardiac microvascular ischemia-reperfusion injury. Basic Res. Cardiol..

[39] Bai M., Lu W., Tan J., Mei X. (2024). Hint2 may be one clinical significance target for patient with diabetes mellitus and reduced ros-induced oxidative stress and ferroptosis by mcu. Horm. Metab. Res..

[40] Deepak K., Roy P.K., Das A., Mukherjee B., Mandal M. (2025). Glucose-6-phosphate dehydrogenase (g6pd) shields pancreatic cancer from autophagy-dependent ferroptosis by suppressing redox imbalance induced ampk/mtor signaling. Free. Radic. Biol. Med..

[41] Jiang Y., Cao Y., Li Y., Bi L., Wang L., Chen Q., Lin Y., Jin H., Xu X., Peng R. (2024). Snp alleviates mitochondrial homeostasis dysregulation-mediated developmental toxicity in diabetic zebrafish larvae. Biomed. Pharmacother..

[42] Jiao Y., Liu X., Shi J., An J., Yu T., Zou G., Li W., Zhuo L. (2024). Unraveling the interplay of ferroptosis and immune dysregulation in diabetic kidney disease: A comprehensive molecular analysis. Diabetol. Metab. Syndr..

[43] Baker M.L., Cantley L.G. (2025). Adding insult to injury: The spectrum of tubulointerstitial responses in acute kidney injury. J. Clin. Investig..

[44] Wu S., Guo M., Wang Y., Zhou Y., Zhang L., Zhou Y., Xing Y., Sun D., Hu X., Ruan Z. (2025). Relationship between podocyte injury and renal outcomes in patients with acute kidney injury: A report from a retrospective study in china. Am. J. Nephrol..

[45] Gong Q., Lai T., Liang L., Jiang Y., Liu F. (2023). Targeted inhibition of cx3cl1 limits podocytes ferroptosis to ameliorate cisplatin-induced acute kidney injury. Mol. Med..

[46] Zhang Z., Ma J., Shi M., Huang J., Xu Z. (2025). Ciapin1 attenuates ferroptosis via regulating pi3k/akt pathway in lps-induced podocytes. BMC Nephrol..

[47] Yau A.A., Murugapandian S., Rizvi A.W., Gaddy A. (2024). Viral nephropathies: Core curriculum 2024. Am. J. Kidney Dis..

[48] Yu P., Jin X., Huang W., Wang J., Zhang S., Ren L., Zhang H., Shi S. (2024). Characterization of immortalized human podocytes infected with lentivirus as an in vitro model of viral infection-associated podocytopathy. Am. J. Clin. Exp. Immunol..

[49] Yang Y.T., Wang X., Zhang Y.Y., Yuan W.J. (2019). The histone demethylase lsd1 promotes renal inflammation by mediating tlr4 signaling in hepatitis b virus-associated glomerulonephritis. Cell Death Dis..

[50] Bian L., Niu Y., Yuan W., Du H., Yang Y. (2024). Hbx promotes glomerular podocyte-induced immune cell responses. Ren. Fail..

[51] Zhan Z., Yang W., Guo W., Wan X., Li J., Zhang Y., Wang B., Liang X., Bai O. (2024). Hbx induces chemoresistance in diffuse large b cell lymphoma by inhibiting intrinsic apoptosis via the nf-kappab/xiap pathway. Mol. Ther. Nucleic Acids.

[52] Zhou H., Wan S., Luo Y., Liu H., Jiang J., Guo Y., Xiao J., Wu B. (2023). Hepatitis b virus x protein induces aldh2 ubiquitin-dependent degradation to enhance alcoholic steatohepatitis. Gastroenterol. Rep..

[53] Yang X., Yu Y., Li B., Chen Y., Feng M., Hu Y., Jiang W. (2023). Bone marrow mesenchymal stem cell-derived exosomes protect podocytes from hbx-induced ferroptosis. PeerJ.

[54] Yuan H., Peng Z., Zhang M., Li H., Lu K., Yang C., Li M., Liu S. (2024). Antagonising yin yang 1 ameliorates the symptoms of lupus nephritis via modulating t lymphocyte signaling. Pharmacol. Res..

[55] Bhargava R., Upadhyay R., Zhao C., Katakam P., Wenderfer S., Chen J., He H., Cummings R., Tsokos M.G., Tsokos G.C. (2025). Aberrant glycosylation of igg in children with active lupus nephritis alters podocyte metabolism and causes podocyte injury. Arthritis Rheumatol..

[56] Liu C., Gan Y.H., Yong W.J., Xu H.D., Li Y.C., Hu H.M., Zhao Z.Z., Qi Y.Y. (2024). Otub1 regulation of ferroptosis and the protective role of ferrostatin-1 in lupus nephritis. Cell Death Dis..

[57] Schindler M., Siegerist F., Lange T., Simm S., Bach S.M., Klawitter M., Gehrig J., Gul S., Endlich N. (2023). A novel high-content screening assay identified belinostat as protective in a fsgs-like zebrafish model. J. Am. Soc. Nephrol..

[58] He X., Yang L., Wang M., Zhang P., Wang R., Ji D., Gao C., Xia Z. (2023). Targeting ferroptosis attenuates podocytes injury and delays tubulointerstitial fibrosis in focal segmental glomerulosclerosis. Biochem. Biophys. Res. Commun..

[59] Braun F., Abed A., Sellung D., Rogg M., Woidy M., Eikrem O., Wanner N., Gambardella J., Laufer S.D., Haas F. (2023). Accumulation of alpha-synuclein mediates podocyte injury in fabry nephropathy. J. Clin. Investig..

[60] Wise A.F., Krisnadevi I.A., Bruell S., Lee H.C., Bhuvan T., Kassianos A.J., Saini S., Wang X., Healy H.G., Qian E.L. (2025). Fabry disease podocytes reveal ferroptosis as a potential regulator of cell pathology. Kidney Int. Rep..

[61] Bellomo F., Pugliese S., Cairoli S., Krohn P., De Stefanis C., Raso R., Rega L.R., Taranta A., De Leo E., Ciolfi A. (2024). Ketogenic diet and progression of kidney disease in animal models of nephropathic cystinosis. J. Am. Soc. Nephrol..

[62] Berlingerio S.P., Bondue T., Tassinari S., Siegerist F., Ferrulli A., Lismont C., Cairoli S., Goffredo B.M., Ghesquiere B., Fransen M. (2025). Targeting oxidative stress-induced lipid peroxidation enhances podocyte function in cystinosis. J. Transl. Med..

[63] Zhao W., Jin G., Sun W., Wu C., Yang Q., Xue L., Ye S. (2025). Empagliflozin alleviates type 2 diabetic renal fibrosis by inhibiting slc7a7-mediated ferroptosis. Diabetol. Metab. Syndr..

[64] Liu X., Zhai X., Wang X., Zhu X., Wang Z., Jiang Z., Bao H., Chen Z. (2025). Nuclear factor erythroid 2-related factor 2 activator ddo-1039 ameliorates podocyte injury in diabetic kidney disease via suppressing oxidative stress, inflammation, and ferroptosis. Antioxid. Redox Signal..

[65] Yang X., Guo N. (2023). Ulinastatin ameliorates podocyte ferroptosis via regulating mir-144-3p/slc7a11 axis in acute kidney injury. Vitr. Cell Dev. Biol. Anim..

[66] Mishima E., Sato E., Ito J., Yamada K.I., Suzuki C., Oikawa Y., Matsuhashi T., Kikuchi K., Toyohara T., Suzuki T. (2020). Drugs repurposed as antiferroptosis agents suppress organ damage, including aki, by functioning as lipid peroxyl radical scavengers. J. Am. Soc. Nephrol..

[67] Zhu X., Lu H., Jia H., Wei X., Xue J., Li W., Zhang J., Wang Y., Yan J., Sun H. (2025). Ferrostatin-1 reduces the inflammatory response of rheumatoid arthritis by decreasing the antigen presenting function of fibroblast-like synoviocytes. J. Transl. Med..

[68] Araoka T., Toyohara K., Ryosaka M., Inui C., Matsuura M., Ma C., Watahiki J., Li Z., Iwasaki M., Watanabe A. (2025). Human ipsc-derived nephron progenitor cells treat acute kidney injury and chronic kidney disease in mouse models. Sci. Transl. Med..

[69] Campa-Carranza J.N., Capuani S., Joubert A.L., Hernandez N., Bo T., Sauceda-Villanueva O.I., Conte M., Franco L., Farina M., Rome G.E. (2025). Immune and angiogenic profiling of mesenchymal stem cell functions in a subcutaneous microenvironment for allogeneic islet transplantation. Adv. Sci..

[70] Huang L.L., Hou Y.Y., Yang J., Liao X.N., Ma J.S., Wang W.C., Quan Y.X., Jiang H.Y., Bai Y.H. (2025). Mitigation of ferroptosis in diabetic kidney disease through mesenchymal stem cell intervention via the smad2/3/mettl3/s1pr1 axis. FASEB J..

[71] Chen Y., Yang X., Feng M., Yu Y., Hu Y., Jiang W. (2024). Exosomal mir-223-3p from bone marrow mesenchymal stem cells targets hdac2 to downregulate stat3 phosphorylation to alleviate hbx-induced ferroptosis in podocytes. Front. Pharmacol..

[72] Zhang Z., Niu L., Tang X., Feng R., Yao G., Chen W., Li W., Feng X., Chen H., Sun L. (2019). Mesenchymal stem cells prevent podocyte injury in lupus-prone b6.mrl-faslpr mice via polarizing macrophage into an anti-inflammatory phenotype. Nephrol. Dial. Transpl..

[73] Liu C., Liu X., Wang Y., Yu H., Li Q., Zheng Y., Fu Y., Yao G., Sun L. (2025). Mesenchymal stromal cells reduce ferroptosis of podocytes by activating the nrf2/ho-1/gpx4 pathway in lupus nephritis. Int. Immunopharmacol..

[74] Shan Y., Lu J., Qian H., Xia Z., Mo X., An M., Yang W., Wang S., Che D., Wang C. (2024). Immobilized protein strategies based on cell membrane chromatography and its application in discovering active and toxic substances in traditional Chinese medicine. Pharmacol. Res..

[75] Li H., Chen H., Gao R., Yin M., Huang F. (2025). Traditional Chinese medicine formulae and Chinese patent medicines for the treatment of diabetic kidney disease: Efficacies and mechanisms. Am. J. Chin. Med..

[76] Gao K., Liu Y., Li K., Liu L., Cai Y., Zhang X., Zhao Z. (2025). Nrf2-mediated ferroptosis is involved in berberine-induced alleviation of diabetic kidney disease. Phytother. Res..

[77] Zhang H., Jiang Y., Song J., Wang S., Lu J., Wei F., Li X. (2025). Urinary exosomes exacerbate diabetic kidney disease by promoting nlrp3 inflammasome activation via the microrna-516b-5p/sirt3/ampk pathway. Am. J. Physiol. Endocrinol. Metab..

[78] Li T., Chen H., Guo Y., Huang M., Liu P., Aikemu A., Mohammadtursun N., Pan X., Yang X. (2025). Nuciferine restores autophagy via the pi3k-akt-mtor pathway to alleviate renal fibrosis in diabetic kidney disease. J. Agric. Food Chem..

[79] Wang H.Q., Wu H.X., Shi W.Q., Yang Y., Lin M., Wang K., Bian C.C., An X.F., Wang T., Yan M. (2024). Triptolide attenuates renal slit diagram to tight junction transition in diabetic kidney disease by regulating nrf2-ferroptosis pathway. Am. J. Chin. Med..

[80] Bhuia M.S., Wilairatana P., Ferdous J., Chowdhury R., Bappi M.H., Rahman M.A., Mubarak M.S., Islam M.T. (2023). Hirsutine, an emerging natural product with promising therapeutic benefits: A systematic review. Molecules.

[81] Hu W., Li M., Sun W., Li Q., Xi H., Qiu Y., Wang R., Ding Q., Wang Z., Yu Y. (2022). Hirsutine ameliorates hepatic and cardiac insulin resistance in high-fat diet-induced diabetic mice and in vitro models. Pharmacol. Res..

[82] Pei Z., Chen Y., Zhang Y., Zhang S., Wen Z., Chang R., Ni B., Ni Q. (2025). Hirsutine mitigates ferroptosis in podocytes of diabetic kidney disease by downregulating the p53/gpx4 signaling pathway. Eur. J. Pharmacol..

[83] Chen J., Ou Z., Gao T., Yang Y., Shu A., Xu H., Chen Y., Lv Z. (2022). Ginkgolide b alleviates oxidative stress and ferroptosis by inhibiting gpx4 ubiquitination to improve diabetic nephropathy. Biomed. Pharmacother..

[84] Jin J., Wang Y., Zheng D., Liang M., He Q. (2022). A novel identified circular rna, mmu_mmu_circrna_0000309, involves in germacrone-mediated improvement of diabetic nephropathy through regulating ferroptosis by targeting mir-188-3p/gpx4 signaling axis. Antioxid. Redox Signal..

[85] Wu W.Y., Wang Z.X., Li T.S., Ding X.Q., Liu Z.H., Yang J., Fang L., Kong L.D. (2022). Ssbp1 drives high fructose-induced glomerular podocyte ferroptosis via activating dna-pk/p53 pathway. Redox Biol..

[86] Wu B., Wang J., Yan X., Jin G., Wang Q. (2025). Cordycepin ameliorates diabetic nephropathy injury by activating the slc7a11/gpx4 pathway. J. Diabetes Investig..

[87] Zhu S., Kang Z., Zhang F. (2024). Tanshinone iia suppresses ferroptosis to attenuate renal podocyte injury in diabetic nephropathy through the embryonic lethal abnormal visual-like protein 1 and acyl-coenzyme a synthetase long-chain family member 4 signaling pathway. J. Diabetes Investig..

[88] Xiong D., Hu W., Han X., Cai Y. (2023). Rhein inhibited ferroptosis and emt to attenuate diabetic nephropathy by regulating the rac1/nox1/beta-catenin axis. Front. Biosci..

[89] Duan J., Pei F., Miao J., Liu S., Tan L., Lu M., Liu Y., Zhang C. (2025). Swietenine improved the progression of diabetic nephropathy through inhibiting ferroptosis via activating akt/gsk-3beta/nrf2 signaling pathway. J. Ethnopharmacol..

[90] Liu S., Zhang F., Bai Y., Huang L., Zhong Y., Li Y. (2024). Therapeutic effects of acupuncture therapy for kidney function and common symptoms in patients with chronic kidney disease: A systematic review and meta-analysis. Ren. Fail..

[91] Yue J.I., Xin-Yuan Z., Yun-Ming X., Zi-Hao Z., Xiao-Hui Y., Xin-Ju L.I. (2024). Acupuncture improve proteinuria in diabetic kidney disease rats by inhibiting ferroptosis and epithelial-mesenchymal transition. Heliyon.

[92] Zhang J., Wu Q., Xia C., Zheng H., Jiang W., Wang Y., Sun W. (2025). Qing-re-xiao-zheng-(yi-qi) formula attenuates the renal podocyte ferroptosis in diabetic kidney disease through ampk pathway. J. Ethnopharmacol..

[93] Zheng H., Tian Y., Li D., Liang Y. (2025). Single-cell multi-omics analysis reveals the mechanism of action of a novel antioxidant polyphenol nanoparticle loaded with stat3 agonist in mediating cardiomyocyte ferroptosis to ameliorate age-related heart failure. J. Nanobiotechnol..

[94] Lange T., Maron L., Weber C., Biedenweg D., Schluter R., Endlich N. (2025). Efficient delivery of small rnas to podocytes in vitro by direct exosome transfection. J. Nanobiotechnol..

